# Role of Extracellular Trap Release During Bacterial and Viral Infection

**DOI:** 10.3389/fmicb.2022.798853

**Published:** 2022-01-26

**Authors:** Bárbara M. Schultz, Orlando A. Acevedo, Alexis M. Kalergis, Susan M. Bueno

**Affiliations:** ^1^Millennium Institute on Immunology and Immunotherapy, Facultad de Ciencias Biológicas, Pontificia Universidad Católica de Chile, Santiago, Chile; ^2^Departamento de Endocrinología, Facultad de Medicina, Pontificia Universidad Católica de Chile, Santiago, Chile

**Keywords:** neutrophil extracellular traps (NETs), virulence factor, bacterial infection, viral infection, extracellular traps (ETs)

## Abstract

Neutrophils are innate immune cells that play an essential role during the clearance of pathogens that can release chromatin structures coated by several cytoplasmatic and granular antibacterial proteins, called neutrophil extracellular traps (NETs). These supra-molecular structures are produced to kill or immobilize several types of microorganisms, including bacteria and viruses. The contribution of the NET release process (or NETosis) to acute inflammation or the prevention of pathogen spreading depends on the specific microorganism involved in triggering this response. Furthermore, studies highlight the role of innate cells different from neutrophils in triggering the release of extracellular traps during bacterial infection. This review summarizes the contribution of NETs during bacterial and viral infections, explaining the molecular mechanisms involved in their formation and the relationship with different components of such pathogens.

## Introduction

Neutrophils, a type of polymorphonuclear cell, are one of the most abundant immune cells in the blood of humans, which increase upon infection with various microbial agents. Neutrophil precursors derived from the bone marrow enter the circulation and are recruited to the infected tissue, where they become fully activated. Activated neutrophils display multi-lobulated nuclei and produce many antimicrobial proteins, different types of granules and reactive chemical species. In addition, these cells present a wide variety of receptors as Pattern recognition receptors (PRRs) that recognize an array of pathogen-associated molecular patterns (PAMPs) and danger-associated molecular patterns (DAMPs). These interactions would enable the recognition of extracellular or intracellular pathogens to trigger responses to clear them (Segal, 2005; Thomas and Schroder, 2013). Furthermore, neutrophils have different mechanisms to develop an efficient bacterial killing, such as phagocytosis, NADPH oxidase-derived reactive oxygen species (ROS), degranulation of cytotoxic components, and antimicrobial peptides (Segal, 2005; Teng et al., 2017). Neutrophils can also release neutrophil extracellular traps (NETs) during microbial infection, a standard mechanism to prevent pathogen spreading during infectious diseases. This review summarizes the current knowledge relative to the mechanism of NET formation during bacterial and viral infections. Furthermore, we also discuss the role of extracellular traps released by other cells, different from neutrophils, which are produced during bacterial infections.

## Neutrophils Extracellular Traps and Netosis

Initial observations described that activated neutrophils were able to generate prominent extracellular structures composed of nuclear chromatin, histones, granular proteins such as neutrophil elastase (NE), myeloperoxidase (MPO), or cathepsin-G, and cytoplasmic proteins such as glycolytic enzymes and catalase, among others (Brinkmann et al., 2004; Urban et al., 2009). Further studies supported the role of NETs as elements able to capture, entrap and kill pathogenic microorganisms (Brinkmann et al., 2004; Papayannopoulos and Zychlinsky, 2009; Pilsczek et al., 2010; Kenny et al., 2017). Roughly, the NETs process begins with the recognition of the microorganism, which activates the NET pathway and allows the disruption of the nuclear and granular membrane, as well as the release of decondensed nuclear DNA into the cytoplasm. This decondensed chromatin mixes with nuclear, granular, and cytoplasmic content and the process ends with the disruption of the plasma membrane and the release of the lattice structure (Fuchs et al., 2007). The cell death process generated by the release of NETs has been denominated NETosis, which is different from other cell death processes described so far. For instance, it is different from apoptosis because it is caspase-independent and no DNA fragmentation is observed, which are hallmarks of the apoptotic process (Fuchs et al., 2007). It is also different from necrosis, because NETosis results in the fragmentation of the nuclear envelopment, which allows the formation of multiple vesicles that mix with the cytoplasm content, a process that does not happen during necrosis (Fuchs et al., 2007). Therefore, NETosis seems to be an innate immune mechanism used to control pathogen spreading by entrapping microorganisms and placing them in direct contact with a high amount of cell-derived antimicrobial molecules (Papayannopoulos and Zychlinsky, 2009). Initially, the release of NETs was thought to be related to the size of the pathogen, because one study shown that small microorganisms, such as single bacteria and unicellular yeast, do not induce NETosis and that the phagocytosis of these unicellular microbes inhibits the release of NETs by sequestering NE (Branzk et al., 2014). However, now it is known that NETs release takes place against fungus (Urban et al., 2005), protozoan (Guimarães-Costa et al., 2009), viruses (Souza et al., 2018), and bacteria (Brinkmann et al., 2004; de Jong et al., 2014). Furthermore, NETs release could be triggered by extracellular or intracellular pathogens (Chen et al., 2018) and, in some cases, the pathogen can generate a vital NETs release, in which the cell continues engulfing the microorganism (Yipp et al., 2012).

Although the function of NETs during microbial infection has a relevant role in pathogen control, it has been described that the overproduction of NETs is also related to tissue damage in several diseases, as such arthritis (Khandpur et al., 2013; Sur Chowdhury et al., 2014), allergies (Bouabe et al., 2011; Hu et al., 2016; Toussaint et al., 2017), systemic lupus erythematosus (SLE) (Kessenbrock et al., 2009; Knight et al., 2012; Wang et al., 2015), and cancer (Cools-Lartigue et al., 2014; Razak et al., 2017; Wang et al., 2021). In the case of inflammatory diseases, it is possible that deficiencies in the mechanisms that prevent excessive tissue damage caused by NETs release are involved in their onset and progression. One of these regulatory mechanisms has been described in M1 macrophages, which degrade DNA in a caspase-activated dependent manner within 24 h post-activation (Nakazawa et al., 2016). Also, it has been described that monocytes-derived macrophages engulf the NETs, a process facilitated by DNase I and opsonization by C1q, without the secretion of pro-inflammatory cytokines after the ingestion (Farrera and Fadeel, 2013).

### Neutrophil Extracellular Trap Induction and Signaling

Neutrophil extracellular traps release was described initially in response to lipopolysaccharide (LPS), interleukin-8 (IL-8), and phorbol myristate acetate (PMA) (Brinkmann et al., 2004; Hakkim et al., 2011). However, further studies have shown that diverse stimuli trigger NETs, such as platelet expressing TLR4 (Clark et al., 2007; Brown and McIntyre, 2011), PAMPs recognition by toll-like receptors (TLR), such as TLR2 (Cacciotto et al., 2016), TLR4 (Funchal et al., 2015), TLR7 (Hiroki et al., 2019), and TLR8 (Lood et al., 2017); calcium ionophores (Pilsczek et al., 2010; Douda et al., 2015), uric acid (Arai et al., 2014), high levels of glucose (Wong et al., 2015; Stoikou et al., 2017; Wang et al., 2018), autoantibodies (Kessenbrock et al., 2009), and interferon (IFN) (Martinelli et al., 2004).

The classical (or suicidal) NETs release, which is activated primarily by PMA, occurs after 3–4 h of stimulation, with the accompanying death of the cell (lytic NETosis). In physiological conditions, the process begins with the recognition of PAMPs or DAMPs by TLR, by receptors of complement system, by Fc-receptors (FcγRIIa and FcγRIIIb) (Chen et al., 2012) or by cytokines (Brinkmann et al., 2004; Garcia-Romo et al., 2011). Then, the Protein Kinase C (PKC) is activated, allowing the activation of the Raf-MEK-ERK pathway and the phosphorylation of a subunit of the NADPH oxidase 2 (NOX2) (Hakkim et al., 2011). Reactive oxygen species (ROS) produced by NOX2 act on the azurophilic granules to release the NE to the cytosol, in a process that requires the function of MPO (Metzler et al., 2014). ROS are also involved in the translocation of NE to the nucleus, promoting the decondensation of chromatin (Papayannopoulos et al., 2010; Metzler et al., 2014). This effect occurs in conjunction with the action of the Peptidyl arginine deiminase 4 (PAD4), an enzyme that citrullinate the histone H3 (Li et al., 2010) and allows NETs release in a process known as NOX2-dependent NETosis (Li et al., 2010). Further, the activity of PAD4 on NETs release is essential for an efficient DNA decondensation, the rupture of the NE granule, the nuclear DNA release into the cytoplasm, and the extracellular NETs release (Thiam et al., 2020).

There is also a NOX2-independent type of NETs release, also known as vital NETosis. This process could be triggered by recognition of LPS by PRRs as TLRs (Pilsczek et al., 2010; Yipp et al., 2012). As soon as 10 min after activation, the extrusion of vesicles loaded with nuclear DNA occurs, without breaking the plasma membrane, with minimal cell lysis and no ROS production (Pilsczek et al., 2010; Chen et al., 2012). The anuclear granulocytes generated -either cytoplasts or motile cytokineplasts- due to vital NETosis retain antimicrobial mechanisms as phagocytosis, transmigration, and chemotaxis (Malawista et al., 1989, 2006). Furthermore, the anuclear neutrophils derived from this process, which are close to 10% of total neutrophils undergoing NETosis, contain intracellular bacteria due to intact plasma membrane and maintain an active phagolysosome, implying that NETs release and phagocytosis can work simultaneously and independently (Yipp et al., 2012). The importance of vital NETs release is that the cell still contributes to the antibacterial mechanisms (Pilsczek et al., 2010; Yipp et al., 2012). However, it is still unknown how long last and how functional these anuclear cells are. Another type of NETs release that retains the survival of the cell is described in neutrophils primed with GM-CSF and stimulated with LPS or with complement components such as C5a, which induce mitochondrial DNA release, a rapid process that depend on ROS (Yousefi et al., 2009; McIlroy et al., 2014). This type of NETs release by viable neutrophils requires glycolytic ATP production for rearrangements of the microtubule network and F-actin (Amini et al., 2018).

Also, NETs release is induced by calcium ionophore or ionomycin, which induce a faster NETs release than the classical NOX2-dependent NETs release and independent of ERK activation (Douda et al., 2015). However, this pathway requires the calcium-activated potassium channel of small conductance 3 (SK channel), which activates mitochondrial ROS production (Douda et al., 2015). In consequence, this process induces the opening of the non-selective mitochondrial permeability transition pore, which results in the accumulation of mitochondrial ROS that causes the activation of NOX2 (Vorobjeva, 2020). Using human neutrophils from healthy controls or from patients with the chronic granulomatous disease (CGD), which lacks NADPH Oxidase, it was demonstrated that NET release can indeed be induced by mitochondrial ROS production (Vorobjeva, 2020).

The last NETs release induction pathway identified so far is mediated by cytosolic LPS derived from intracellular Gram-negative pathogens, such as *Salmonella enterica* serovar Typhimurium (Chen et al., 2018). This stimulus activates the non-canonical inflammasome, which is a caspase-11-dependent pathway, and triggers the neutrophil gasdermin-D (GSDM-D)-dependent death (Chen et al., 2018). In this case, the action of ROS or PAD4 is not required, because GSDM-D can directly generate pores in the nuclear and plasma membranes (Chen et al., 2018).

It is essential to mention that not all stimuli activate the specific proteins discussed above. Although five different stimuli induce NETs production, killed bacteria and degraded proteins activate different pathways as compared to NOX2-dependent NETosis (Kenny et al., 2017). For instance, *Candida albicans* and group B of *Streptococcus* (GBS) induce NETs independently of histone citrullination mediated by PAD4 (Kenny et al., 2017; Guiducci et al., 2018). In another study, *Leishmania amazonensis* induced both types of NETs release: the classical NOX2-dependent NETosis (which is dependent on the action of PAD4, but independent of MPO) and the early/rapid NETosis (which is ROS and NE-independent, but dependent on the activity of PAD4) (Rochael et al., 2015). All these studies suggest that the NETosis process is not activated just by one or two pathways but depends on the nature of the stimulus and can be very diverse in terms of activation.

It has been described that other factors produced due to the host immune response activation can also induce NETs during a bacterial infection. As an example, it has been described that platelets can recognize Gram-negative and -positive bacteria and other stimuli through TLR-4, inducing the adhesion to neutrophil and NETs release (Clark et al., 2007). Accordingly, elimination of platelets or the inhibition of TLR4 expressed by platelets substantially reduces NETs release (Clark et al., 2007). The importance of platelets is observed when the bacteria induce virulence factors that promote apoptosis of these cells, causing thrombocytopenia and generating a more severe infection that affects the immune response of the neutrophils (Kraemer et al., 2012). Other factors produced during immune response activation that cause NETs release in a ROS- dependent manner are of pro-inflammatory cytokines such as TNFα, IL-1β, or IL-8 (Keshari et al., 2012) and macrophage Migration Inhibitory Factor (MIF) secreted by red blood cells during *Plasmodium* infection, which induce NETosis in a C-X-C chemokine receptor type 4 (CXCR4) dependent manner (Rodrigues et al., 2020).

## Other Immune Cells That Produce Extracellular Traps

Although NETs are the most studied Extracellular Traps (ET), other immune cells are also able to produce this kind of structures, such as eosinophils (Ueki et al., 2013), basophils (Yousefi et al., 2015), macrophages (Aulik et al., 2012), and mast cells (Naqvi et al., 2017).

### Eosinophils Extracellular Traps

Eosinophils extracellular traps (EETs) are released similarly to NETs. They are triggered due to activation by bacteria (Yousefi et al., 2008; Gevaert et al., 2017), fungi (Muniz et al., 2018; Omokawa et al., 2018), and by PMA and calcium ionophore stimulation (Ueki et al., 2013, 2016). In addition, it has been described that EETs contain entire eosinophil granules and granule-derived proteins (Mukherjee et al., 2018). Furthermore, mitochondrial DNA-derived EETs has also been reported in response to LPS stimulation on previously primed cells in *in vitro* experiments, which did not involve cell death (Yousefi et al., 2008). However, other studies have shown that EETs can also be produced by nuclear DNA, and ROS production dependent on NOX activation, in a similar pathway to the lytic NETosis (Ueki et al., 2013). The presence of EETs has been reported during allergies (Dworski et al., 2011), respiratory tract disease (Cunha et al., 2014; Ueki et al., 2016; Echevarría et al., 2017), and skin disease (Simon et al., 2011; Morshed et al., 2012).

### Basophils Extracellular Traps

The release of basophils extracellular traps (BETs) has not been deeply studied. However, it has been observed in activated cells in response to Gram-positive (Morshed et al., 2014) and -negative bacteria (Yousefi et al., 2015). It has been described that ETs derived from mice or human basophils have the capacity to entrap *Escherichia coli* and *Staphylococcus aureus* (Yousefi et al., 2015), equivalent to NETs (Brinkmann et al., 2004). Another *in vitro* study performed in basophils derived from mice and humans reported that BETs are released in a mitochondrial ROS-dependent manner, without activation of NOX, and are also composed of mitochondrial DNA (Yousefi et al., 2015). This process can take place as rapidly as 5 min post-stimulation (Yousefi et al., 2008) with the concomitant cell survival of human and mouse primed basophils (Morshed et al., 2014). Lastly, BETs release was reported *in vivo* studies during *N. brasiliensis* infection in mice and inflammatory skin diseases in the human epidermis (Morshed et al., 2014).

### Macrophages and Monocytes Extracellular Traps

Macrophages and monocyte extracellular traps (METs) have been studied in different types of cells, such as RAW264.7, human alveolar macrophage, murine peritoneal macrophages, and bovine monocytes (Chow et al., 2010; Doster et al., 2018). Experiments performed in *in vitro* and *in vivo* models have described that TNF-α is an inducer of ETs in RAW264.7 macrophages. The concomitant presence of citrullinated histones suggests that ETosis in macrophages is mediated by PAD-2 (Mohanan et al., 2013). Various distinct stimuli can induce METs, including Gram-negative (Webster et al., 2010; Liu et al., 2014) and -positive bacteria (Chow et al., 2010; Hellenbrand et al., 2013; Shen et al., 2016), parasites (Muñoz-Caro et al., 2014; Reichel et al., 2015), fungi (Liu et al., 2014; Halder et al., 2017; Loureiro et al., 2019), PMA (Chow et al., 2010), and TNF-α (Mohanan et al., 2013), leading to cell death. Monocyte-derived macrophages from human peripheral blood have also been described to release METs after the stimulation with IFN-γ during infection with *Mycobacterium tuberculosis* (Wong and Jacobs, 2013). *Escherichia coli* also induces the release of METs, composed of nuclear and mitochondrial DNA, histones, MPO, and lysozyme (Liu et al., 2014), independently of ROS production by NOX (Liu et al., 2014). Like NETs, METs can be produced by different molecular pathways, dependent or independent of ROS and caspase-1, in human monocytes derived from peripheral blood when infected with *Escherichia coli* and *Klebsiella pneumoniae* infection (Webster et al., 2010). METs have been also observed in bone marrow-derived macrophage and J774A.1 macrophages infected with a mutant strain of *Salmonella enterica* serovar Typhimurium, showing that METs can kill and entrap at least 10% of the initial inoculum (Mónaco et al., 2021). However, more studies are necessary to determine the different pathways that induce METs release and to identify similarities between NETs and METs, because the ETs from macrophages depends on the specie, the state of differentiation, microenvironment, and state polarization (Doster et al., 2018).

### Mast Cells Extracellular Traps

The release of mast cells extracellular traps (MCETs) was first described in response to PMA, with ROS production by NOX (Von Köckritz-Blickwede et al., 2008; Campillo-Navarro et al., 2018). MCETs are composed of nuclear DNA, histones, and granular proteins, such as tryptase and cathelicidin AMP LL-37 (Campillo-Navarro et al., 2017), which suggest that MCETs and NETs are produced in a similar manner (Von Köckritz-Blickwede et al., 2008). It has been shown that although mast cells cannot phagocytose *Streptococcus pyogenes*, the release of MCETs allows the growth inhibition of the bacteria (Von Köckritz-Blickwede et al., 2008). Furthermore, heat-killed *Mycobacterium tuberculosis* can induce the release of MCETs after 2 h of stimulation; however, the live pathogen can modulate the release of these ETs (Campillo-Navarro et al., 2018). MCETs are released in a ROS-dependent manner in the case of *Listeria monocytogenes* (Campillo-Navarro et al., 2017).

## Role of Neutrophil Extracellular Trap During Bacterial Infection

The contribution of NETs during bacterial infection is not completely clear. It was initially thought that it promoted the clearance of bacteria by facilitating the entrapping and killing of these pathogens (Riyapa et al., 2012). However, NETs release has a bacteriostatic rather than a bactericidal effect because it mainly affects the growth of the bacteria and eventually could aid the killing (Menegazzi et al., 2012). Nevertheless, the DNA exerts antimicrobial properties by cation chelation and the disruption of the cell membrane (Halverson et al., 2015). Furthermore, experiments in primary human neutrophils showed that NETs could entrap bacteria, including *Pseudomonas aeruginosa* (*P. aeruginosa*) and *Staphylococcus aureus* (*S. aureus*), without killing them but affecting the ability of the complement to kill them (Azzouz et al., 2018). Also, the condition in which the NETs are produced affects the antimicrobial properties, observing that NETs in static conditions present fewer killing abilities than the NETs released in dynamic conditions (Azzouz et al., 2018).

On the other hand, NETs release could result in collateral effects due to the production of antimicrobial components that can lead to exacerbated inflammation, causing tissue damage (Xu et al., 2009). However, it has been described that the granular proteins with bactericidal activities released within the NET act mainly as a regulator of inflammation due to the action on different cytokines, rather than as a bactericidal mechanisms (Clancy, 2018).

**FIGURE 1:**
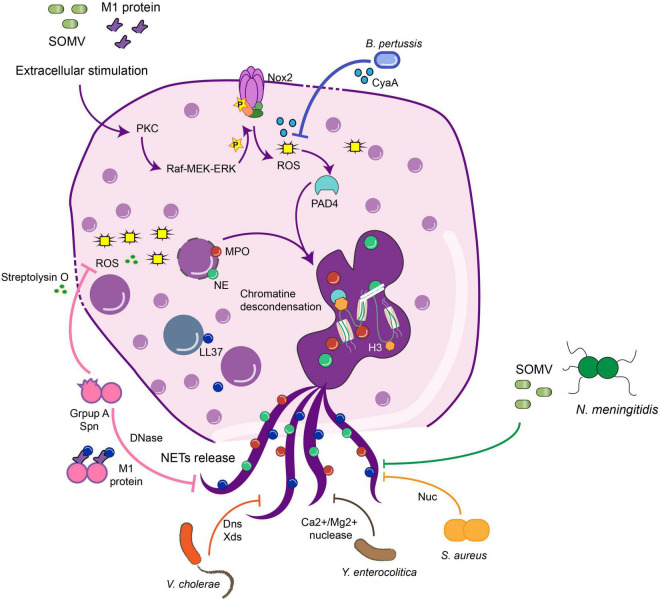
Bacteria virulence factors that avoid NETs release against bacterial infection. Bacteria have evolved to develop different virulence factors to avoid the function of NETs, inhibiting different steps in the pathways required for the NETs release. *B. Pertussis* or GAS inhibit the action of ROS production by streptolysin O during the NETs pathway, which in the end inhibits the release of the structure. Nucleases are the main virulence factor shared among the bacteria which dismantle the NETs structure, and in this sense, the bacteria can disseminate and generate the disease. GAS, Group A *Streptococcus*; MPO, myeloperoxidase; NE, neutrophil elastase; SOMV, small vesicles from the outer membrane into the environment; PAD4, peptidylarginine deiminase 4; Nuc, nuclease; LL37, cathelicidin.

Notably, while almost all bacteria can induce ETs, several microorganisms have evolved molecular strategies to inhibit this host mechanism of defense to promote microbial proliferation and dissemination (Malachowa et al., 2013; Seper et al., 2013; Storisteanu et al., 2017). Evasion strategies can be due to the inhibition of NET release by down-regulating host inflammatory responses, the degradation of NETs using pathogen-derived DNases, and/or by the resistance to the microbicidal components of NETs (Halverson et al., 2015; Storisteanu et al., 2017; Figure 1). In this section, we will review some examples of virulence factors that induce the NETs, which can favor or not the clearance, and some evasion strategies used to avoid the antimicrobial mechanisms and, in some cases, take advantage of this immune response. These evasion mechanisms have been summarized in Table 1.

**TABLE 1:** Virulence factors that interfere with NET function during bacterial infection.

	NET inhibition	Type of inhibition of NET function	References
*Pseudomonas aeruginosa*	Dnase (eddB), phosphatase (eddA)	2	Rada et al., 2013; Wilton et al., 2018
	Biofilm formation	3	Thanabalasuriar et al., 2019
	Pyocyanin	1	Rada et al., 2013
*Mycobacterium tuberculosis*	Probably the level of lipids of it envelops	3	Liu et al., 2019; Sun et al., 2020
	ESAT-6 protein by the ESX-1 type	1	Francis et al., 2014
*Staphylococcus* *aureus*	Biofilm formation	3	Malachowa et al., 2013; Bhattacharya et al., 2018
	Eap, DNA binding protein, Nuc, adenosin synthase	2	Chavakis et al., 2005; Thammavongsa et al., 2013; Eisenbeis et al., 2018
	Leukotoxin GH	1	Malachowa et al., 2013
*Bordetella* spp.	ACT and CyaA	1	Eby et al., 2014; Gorgojo et al., 2017
*Streptococcus* spp.	Sda1	3	Lauth et al., 2009; Buchanan et al., 2006; Moon et al., 2016
	SpnA	3	Chang et al., 2011
	endA	3	Beiter et al., 2006; Zhu et al., 2013
	Capsule	3	Wartha et al., 2007
	*dlt* operon	3	Wartha et al., 2007
	Streptolysin O	1	Uchiyama et al., 2015
	M1 toxin	1	Lauth et al., 2009
*Yersinia* *enterocolitica*	Nuclease	2	Möllerherm et al., 2015
*Vibrio cholerae*	Dns and Xds	2	Seper et al., 2013
*Mycoplasma pneumoniae*	Mpn491	2	Yamamoto et al., 2017
*Neisseria* *meningitidis*	Nuc	2	Lappann et al., 2013
	Phosphoethanolamine transferase	3	Lappann et al., 2013
	ZnuD	2	Lappann et al., 2013
	SOMVs	3	Lappann et al., 2013
*Burkholderia pseudomallei*	TTSS and capsule polysaccharide I	1	Riyapa et al., 2012

*Inhibition of NET release by down regulating the host inflammatory response^1^; Degradation of NETs using pathogen derived DNases^2^; Bacterial virulence factors that evade NETs^3^.*

### Pseudomonas aeruginosa

*Pseudomonas aeruginosa* is an encapsulated, Gram-negative bacterium associated with severe illnesses in healthy and individuals with comorbidities and an important cause of nosocomial infection in cystic fibrosis patients (Davies, 2002). Pyocyanin, a redox-active pigment secreted to the airways by the biofilm, is associated with an increase in oxidative stress and the inflammation generated during the disease (Rada et al., 2013). Also, this virulence factors increases the induction of NETs by the NOX2-dependent pathway (Rada et al., 2013). However, NETs extrusion does not have any effect in entrapping or killing the bacteria but in decreasing the functionality of the lungs and increasing the inflammatory conditions found in patients with cystic fibrosis (Rada et al., 2013).

The sputum of cystic fibrosis patients presents a large amount of DNA because neutrophils is one of the main types of immune cells recruited to the airways and the NETs release by these cells allow the characteristic sputum’s mucus structure (Manzenreiter, 2012). In this sense, it has been observed that the concentration of extracellular DNA in the sputum generate the lysis of the bacteria (Halverson et al., 2015). However, *P. aeruginosa* contains virulence factors that allow the degradation of the NETs structure, which involves an operon encoding two DNA-modifying type II secreted enzymes: a DNase (*eddB*) and a phosphatase (*eddA*) (Wilton et al., 2018). These two enzymes work together at degrading the extracellular DNA: the phosphatase acts on the phosphodiester backbone of the DNA, removing the phosphates and altering the function, but no the structure of the DNA (neutralizes its cation-chelating, antimicrobial activity), while the DNase disassembles the NETs and promotes bacterial survival (Wilton et al., 2018). This allows the bacteria to tolerate the ETs produced by the neutrophils, in addition to the formation of the biofilms that avoid the NETs antimicrobial function. This role of the NETs in the consistency of the sputum has led to the evaluation of rhDNases used as treatment (Guichard et al., 2018).

In cystic fibrosis patients, *P. aeruginosa* can produce keratitis caused by a biofilm formed in the outer eye surface (Saraswathi and Beuerman, 2015; Naimie et al., 2016). In a mice model of keratitis caused by *P. aeruginosa*, it was observed that the Type Three Secretion System (TTSS) and the bacterial Psl exopolysaccharide contribute to biofilm formation (Jabalameli et al., 2012; Naimie et al., 2016) and the release of NETs (Thanabalasuriar et al., 2019). In this model, NETs production allows the maturation of the biofilm formation and the inhibition of the dissemination to the brain. However, NETs formation generates a severe local ulcer in the eye without killing the pathogen (Thanabalasuriar et al., 2019).

### Mycobacterium tuberculosis

*Mycobacterium tuberculosis* (Mtb) is the causative agent of tuberculosis, a chronic infectious lung disease that affects over one-third of the global population and causes 8 million new cases per year (Theodor, 2013; Ramazanzadeh et al., 2015). Mtb secrete the protein ESAT-6 by the ESX-1 type VII secretion system, increasing Ca^2 +^ influx inside the cell, activating calpain, a cysteine protease, which finally allows the release of NETs structures through a pathway similar to the activation produced by ionomycin (Francis et al., 2014). This mechanism contributes to lung pathology and generates an environment more permissive to infection (Francis et al., 2014). Furthermore, in neutrophils obtained from human alveolar lining fluid, ETs structures fail to kill Mtb but contribute to reducing the bacterial dissemination (Arcos et al., 2015).

It is also known that the bacteria *Mycobacterium bovis* or Bacillus Calmette Guerin (BCG), which is currently used as a vaccine against *Mycobacterium tuberculosis*, can trigger NET formation (Liu et al., 2019; Sun et al., 2020). This bacterium is currently used to induce protection against heterologous antigens as well (Claudia et al., 2015; Céspedes et al., 2017; Goulart et al., 2017; Soto et al., 2018; Covián et al., 2019) and is also used as a immunotherapy in different diseases in humans. The NETs pathway activated by BCG depends mainly on ROS production (Liu et al., 2019; Sun et al., 2020). Furthermore, it was also shown that NETs entrap but do not kill bacteria, which may be due to the high lipid levels of the mycobacterial envelope that impair NET-mediated killing (Liu et al., 2019; Sun et al., 2020).

### Staphylococcus aureus

*Staphylococcus aureus* is a Gram-positive bacterium usually part of the normal microbiota (Krismer et al., 2017). However, this bacterium can act as an opportunistic pathogen and eventually be the causative agent of significant systemic disease due to the activity of several virulence factors. It was observed in human neutrophils that *S. aureus* induces NETs due to a leukotoxin GH (LukGH), which generates NETs release and entrap but does not kill the bacteria (Malachowa et al., 2013). In a porcine chronic burn model, *S. aureus* biofilms -in opposite to single-cell populations- promote the formation of NETs through the combined action of leukocidins Panton-Valentine leukocidin (PVL) and γ-hemolysin AB (Bhattacharya et al., 2018) without the avoidance of bacterial dissemination (Malachowa et al., 2013). Through this response, *S. aureus* persisted because the antimicrobial activity of NETs was ineffective at eliminating the bacteria associated with the biofilm (Bhattacharya et al., 2018; Speziale and Pietrocola, 2021).

*Staphylococcus aureus* also produce different enzymes to interfere with the antimicrobial property of the extracellular DNA, such as a DNA binding protein, the extracellular adherence protein (Eap) (Chavakis et al., 2005), and a nuclease (Nuc). These enzymes allow the escape from the NETs structure, delay the bacteria clearance and increase the mortality caused by the infection. Also, this nuclease is related to the persistence of the bacteria in cystic fibrosis patients (Berends et al., 2010; Herzog et al., 2019). Eap binds to linearized extracellular DNA, aggregates this structure, and interferes with the antimicrobial and trapping function of NETs structure in human-derived neutrophils (Chavakis et al., 2005; Eisenbeis et al., 2018). On the other side, the adenosine synthase (Thammavongsa et al., 2013) in conjunction with a nuclease Nuc are required to generate deoxyadenosine (dAdo) from dsDNA derived from the NETs release in human-derived neutrophils, inducing caspase-3-mediated death on macrophages that are recruited to the site of infection (Figure 1; Thammavongsa et al., 2013).

### *Bordetella* spp.

*Bordetella pertussis* (*B. pertussis*) is a Gram-negative bacterium and the causative agent of whooping cough, causing approximately 151,000 cases globally in 2018, according to the World Health Organization (WHO). This bacterium expresses several virulence factors as pertussis and adenylate cyclase toxins (Ladant and Ullmann, 1999; Mooi et al., 2009). The adenylate cyclase toxin (ACT) prolongs the life span of human-derived neutrophils and inhibits the release of NETs by increasing cAMP levels and inhibiting intracellular ROS production (Eby et al., 2014). *Bordetella parapertussis* also generates whooping cough (Watanabe and Nagai, 2004; Bouchez and Guiso, 2015) and produce an adenylate cyclase enzyme, CyaA, that is released to the extracellular medium and inhibits the ROS production generated by NOX (Gorgojo et al., 2017), inhibiting the NET induction in human-derived neutrophils (Figure 1). However, NETs induced by these bacteria can trap and kill bacteria because they fail to express other virulence factors to dismantle the structure (Gorgojo et al., 2017).

### *Streptococcus* spp.

Group A *Streptococcus* (GAS) is a group of Gram-positive, β-hemolytic bacteria, part of the normal microbiota that can generate between 10,649 to13,434 cases of invasive GAS infections that occur in the United States annually (Stevens, 1992; Deutscher et al., 2011; Nelson et al., 2016). In human neutrophils, GAS expresses the M1 exotoxin, a virulence factor, which induces ETs in neutrophils and mast cells by associating with fibrinogen and forming a complex that stimulates neutrophils (Lauth et al., 2009). However, these ETs do not kill the pathogen because the M1 exotoxin allows the pathogen’s survival in the presence of cathelicidin and antimicrobial peptides (Lauth et al., 2009). Besides this, GAS expresses a DNase Sda1, which promotes the degradation of the NETs (Buchanan et al., 2006; Moon et al., 2016). *Streptococcus pyogenes* is the main species that belongs to GAS. Besides Sda1 (Buchanan et al., 2006), it produces another nuclease, SpnA, that is not secreted but is anchored to the cell wall and allows bacteria survival in human blood and resist NETs killing (Chang et al., 2011). Also streptolysin O, a pore-forming toxin, induces eukaryotic cell lysis (Uchiyama et al., 2015) due to a decrease in the oxidative burst and, consequently, inhibits the release of NETs and the extracellular killing (Uchiyama et al., 2015) allowing bacteria survival in the bloodstream.

*Streptococcus pneumoniae* (*S. pneumoniae*) is an alpha-hemolytic bacterium and the leading cause of pneumonia worldwide, mainly in children, that caused 294,000 deaths during 2015 (Wahl et al., 2018). It has been described that the induction of NETs in this disease has been correlated with an adverse outcome in community-acquired pneumonia (CAP) (Gray, 2018). However, in a mice model of infection, it was observed that although NETs can entrap *S. pneumoniae* (Figure 1), it fails to kill this pathogen due to the expression of endA, a bacterial cell-bound DNase. EndA destroys the NETs and promotes the spreading of bacteria from the upper airways to the lungs and bloodstream, promoting a more invasive disease (Beiter et al., 2006; Zhu et al., 2013). The *S. pneumoniae* capsule also contributes to avoiding the bacterial entrapping by NETs (Wartha et al., 2007). Due to the operon *dlt* that produces the modification of lipoteichoic acids, which introduce positive charge into alanine amino acid residues, which then caused electrochemical repulsion of antimicrobial proteins present in NETs (Wartha et al., 2007). This molecular mechanism contributes to bacterial resistance to the killing by NETs (Wartha et al., 2007). Importantly, it has been described that IL-10 production by neutrophils in C57BL/6 mice can modulate the lung injury induced by *S. pneumoniae* infection (González et al., 2021). It has been described that IL-10 can inhibit the TLR7/8 activation pathway, which prevents the generation of ROS and the translocation of NE to the nucleus, decreasing the NETs release (Saitoh et al., 2012), but it is unknown whether IL-10 producing neutrophils are still able to produce NETs.

### Klebsiella pneumoniae

*Klebsiella pneumoniae* (*K. pneumoniae*) is a Gram-negative bacteria found in the nasopharynx and the intestinal tract. It is the most relevant species for humans of the genus *Klebsiella* spp. and a significant cause of nosocomial infection, responsible for severe diseases such as septicemia, pneumonia and urinary tract infections (Podschun and Ullmann, 1998). In the United States, the infection caused by Carbapenem-resistant Enterobacteriaceae (CRE) produce up to 2.93 cases per 100,000 people (Guh et al., 2015). *CRE* sequence type 258 (CRKP-ST258) is a multidrug-resistant strain that has spread worldwide, which evades the neutrophil immune response, preventing intracellular killing and NETs release in neutrophils derived from human (Castillo et al., 2019) and mice (Peñaloza et al., 2020). In human neutrophils, it was described that inhibition of NETs release was due to the avoidance of ROS production, produced to in part to the polysaccharide of the LPS (Castillo et al., 2019). In mouse neutrophils, no differences in ROS or MPO was observed when compared to a non-pathogenic *K. pneumoniae*, but differences in the acidification of the phagolysosome was described, which affects the functionality of MPO (Peñaloza et al., 2020). Another *in vitro* study performed in human-derived neutrophils showed that *K. pneumoniae* carbapenem-resistant affects the release of NETs due to the mobilization of primary granules due to a non-soluble virulence factor (Birnberg-Weiss et al., 2021).

### Other Gram-Negative Bacteria

*Yersinia enterocolitica* is the causative agent of yersiniosis auto-limited gastroenteritis (Marks et al., 2018), producing 9.7 cases each 100,000 people per year, being children between 6 and 11 months the most affected (Yagüe-Muñoz et al., 2019). Three different serotypes (O:3, O:8, and O:9) were tested for induction of NETs release in human-derived neutrophils and all of them induce and degrade NETs by the action of Ca^2+^/Mg^2+^-dependent NET-degrading nuclease (Figure 1; Möllerherm et al., 2015). Secretory diarrheal disease caused by *Vibrio cholerae*, the causative agent of a previously considered non-inflammatory disease, has recently been shown to recruit a high number of neutrophils (Queen and Satchell, 2012). Human-derived neutrophils in direct contact with *Vibrio* release NETs in an oxidative burst-dependent fashion and can kill the bacteria (Seper et al., 2013). Nevertheless, at the same time, bacteria induce two extracellular nucleases: Dns and Xds, which enhance pathogen dissemination (Figure 1; Seper et al., 2013). *Mycoplasma pneumoniae* causes atypical pneumonia and produces an extracellular nuclease, Mpn491, that requires Mg^2+^ to degrade the NETs structure in *in vitro* and *in vivo* models (Yamamoto et al., 2017).

*Neisseria meningitidis* (meningococci) is a Gram-negative bacterium that can cause severe septicemia in children and is a restricted human pathogen. *Neisseria* also presents a putatively secreted thermonuclease denominated Nuc, which induces and degrades NETs from human-derived blood, contributing to the escape and the avoidance of the killing of the pathogen (Lappann et al., 2013). Meningococcus display at least three different mechanisms to avoid NETs killing: (1) the lipid A modification of LPS with a phosphoethanolamine transferase is crucial for the survival of *Neisseria meningitidis* in the presence of NETs *in vitro*, due to this modification, bacteria are protected from the action of the cathepsin-G antimicrobial peptide; (2) it produces an outer membrane receptor ZnuD, which is crucial to uptake the Zn^2+^ and promote the nutritional resistance in the environment induced by the NETs; (3) it secrete small vesicles from the outer membrane into the environment (SOMVs), which have been identified as the inducers of NETs release and also bind to the NETs structure to reduce its bacteriostatic effect (Lappann et al., 2013; Figure 1).

*Burkholderia pseudomallei* is a Gram-negative bacterium and the causative agent of melioidosis, a zoonotic infection leading to lung, localized or systemic infection. It is a critical pathogen in diabetic patients, and it is estimated that exist 165,000 human melioidosis cases per year, of which 89,000 people die with a case fatality rate of more than 50% (Chanchamroen et al., 2009; Limmathurotsakul et al., 2010; Birnie et al., 2019). This bacterium triggers the induction of NOX2-dependent NETs released in human- and mouse-derived neutrophils, and in addition to entrapping bacteria, NETs can significantly reduce the initial inoculum (Riyapa et al., 2012). However, the TTSS and capsular polysaccharide-1 expressed by these bacteria can regulate the proportion of NETs released, possibly by regulating the oxidative burst (Riyapa et al., 2012).

*Leptospira* spp. is an important cause of zoonotic infection, which can generate rapid bloodstream dissemination and affect mainly the kidney function, and in this manner, the carrier disseminates the infection (Scharrig et al., 2015). It was shown that *Leptospira interrogans* serovar Copenhagen strain Fiocruz L1-130 (LIC) induces NETs released by human- and mouse-derived neutrophils, which entraps and kills bacteria, decreasing the CFU content. However, some pathogenic strains can degrade the dsDNA structure, implying that the NETs function depends on the infecting bacterial strain (Scharrig et al., 2015).

## Contribution of Neutrophil Extracellular Trap to Viral Infections

In addition to the widely described role of bacteria in triggering NETs release, increasing evidence indicates that viruses can also promote NET formation (Jenne et al., 2013; Souza et al., 2018). Current data suggest that PRRs expressed on the surface or internal compartments of neutrophils, such as endosomes, play a crucial role in triggering NETs release (Saitoh et al., 2012). Other studies suggest that, as observed for bacteria, viruses may counteract the mechanisms involved in triggering NETs release during infection (Martinez et al., 1996). It is also documented that mechanisms underlying NET release induced by viruses may differ mechanistically depending on the specific pathogen involved (Muraro et al., 2018; Chan et al., 2020). Along these lines, it has been described that NET release may either promote or prevent the viral-induced pathology (Jenne et al., 2013; Al-Anazi et al., 2020).

**FIGURE 2:**
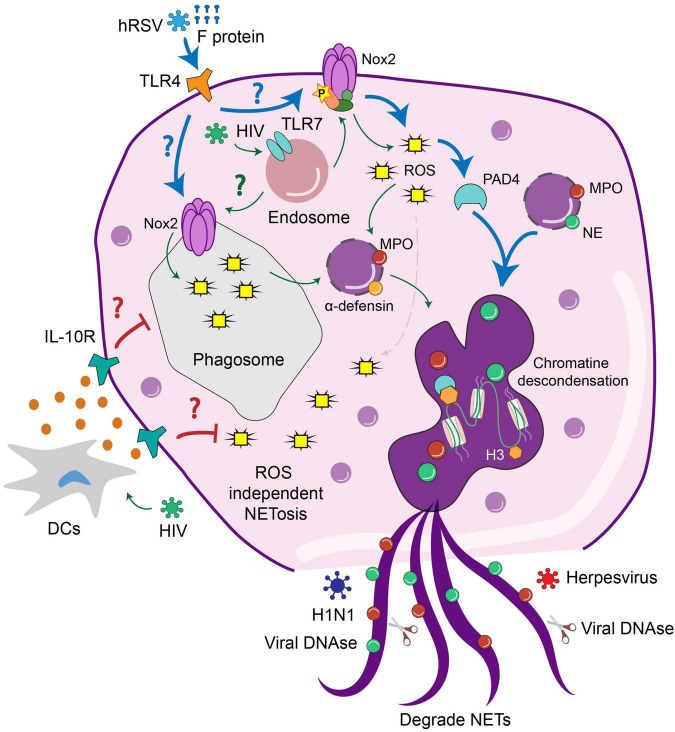
The release and activity of NETs is modulated by viral infection. Interaction of neutrophils with different viruses activates extracellular or intracellular pathways that lead to the NET formation. HIV triggers TLR7 signaling in the endosomes of neutrophils leading to the production of ROS and subsequently NETs release. HIV infection of DCs triggers the production of IL-10, which suppresses the formation of NETs and may allow pathogen spreading. HRSV infection triggers TLR4 signaling at the cell surface, which results in ROS-dependent NETs release. In the case of H1N1, this virus triggers ROS independent NETs release which may prevent pathogen spreading. In contrast, the activity of DNases from Herpesviruses can degrade NETs to allow viral dissemination.

In the case of Human Immunodeficiency Virus-1 (HIV-1), it has been documented that NETs promote pathogen clearance through the concerted action of MPO, α-defensin, and histones (Saitoh et al., 2012). In this work, cultivated neutrophils were exposed to HIV and the results suggest that NETs release induced by infection occurs through the engagement of endosome-expressed TLR7 and TLR8, which induce ROS-dependent NET formation (Figure 2). Therefore, in this case, NET formation benefits the infected host to prevent pathogen spreading (Saitoh et al., 2012). Remarkably, HIV-1 counteracts this response by inducing the production of IL-10 by DCs, which suppresses the ROS-dependent response that results in an impaired NET-dependent HIV-1 elimination.

Another example of a protective role of NETs during viral infection is the case of the myxoma virus (MYXV) (Jenne et al., 2013). This oncolytic virus is characterized by its ability to infect rabbits and kill human and murine cancer cells (Lun et al., 2005). Therefore, it has been proposed as a viral-based therapy for cancer (Rahman and McFadden, 2020). In the mice model, intravenous infection with MYVX induces massive recruitment of neutrophils and platelets to the liver vasculature (Jenne et al., 2013). At this site, the interaction of both subsets of cells promotes the release of NETs that can protect host cells from MYVX infection, and this protective effect was reversed by DNase treatment (Jenne et al., 2013). These results highlight the role of extracellular DNA in preventing viral dissemination (Jenne et al., 2013). According to this notion, it has also been shown that viral proteins with DNase activity derived from Herpesviruses can also degrade NETs, thereby preventing the formation of NETs and promoting viral spreading (Martinez et al., 1996).

Influenza is another respiratory virus that circulates worldwide and can trigger NETs release (Ashar et al., 2018; Zhu et al., 2018; Chan et al., 2020). Annual influenza cases lead to extensive mortality, especially in people older than 65 years. According to a recent study, it was estimated that influenza infection accounts for 4.0–8.8 deaths per 100,000 individuals annually (Iuliano et al., 2018). Sudden onset of fever, headache, sore throat, and a runny nose develops upon infection. Illnesses range from mild to severe and can lead to the death of infected individuals (Ryu and Cowling, 2020). Patients with severe influenza showed elevated plasma NET release, measured as the level of cell-free DNA and DNA-MPO complexes (Zhu et al., 2018). In addition, isolated neutrophils from these subjects released higher amounts of MPO-DNA complex in response to IL-8 or LPS (Zhu et al., 2018). Interestingly NETs from H7N9 and H1N1 patients increased the permeability of alveolar epithelial cells, and, consequently, NET production was positively correlated with acute inflammation (Zhu et al., 2018). Together, this data indicates that high levels of NETs correlate with influenza severity. Thus, evaluation of NETs in plasma could be an excellent strategy to predict the prognosis of IAV patients (Zhu et al., 2018).

Histones present in the lungs of IAV infected mice have been shown to induce cytotoxicity on cultured human lung epithelial cells (Ashar et al., 2018). Furthermore, histones also bind to platelets within thrombi in infected mouse lungs (Ashar et al., 2018). Nasal aspirates from influenza-infected patients also have elevated levels of extracellular histones, which may serve as a clinical marker of pulmonary injury (Ashar et al., 2018). *In vitro* studies showed that histones inhibited influenza growth. However, in the mice model, *in vivo* treatment with histones did not yield antiviral effects and instead increased lung illness (Ashar et al., 2018). The blockade of histones with anti-histone antibodies caused a significant reduction of lung pathology in lethal influenza–challenged mice and enhanced protection when co-administered with the antiviral oseltamivir (Ashar et al., 2018). These data highlight the pathogenic effects of extracellular histones in pulmonary injury during influenza infection. These findings suggest that targeting histones represents a novel therapeutic strategy for treating influenza pneumonia (Ashar et al., 2018).

Another study described that NETs release is triggered only by some IAV specific strains (Chan et al., 2020). For example, it is indicated that the H5N1 strain fails to stimulate NETs release, whereas H1N1 infection stimulates NET production by isolated human neutrophils (Chan et al., 2020). Furthermore, it is also thought that infection with H5N1 caused a more severe disease than H1N1 infection (Chan et al., 2020), which opens the question of whether there are other innate immune responses rather than NETs release that can account for more severe disease (Chan et al., 2020). The same study suggested that NET production induced by H1N1 is not dependent on the NOX-produced ROS (Chan et al., 2020). Consistent with this notion is the observation that neutrophils exposed to the NOX inhibitor diphenyleneiodonium (DPI) were able to produce NETs in response to an H1N1 challenge (Chan et al., 2020). Thus, as observed for some bacteria, such as *S. aureus*, the possibility that NET release occurs independently of ROS production is also described for viruses (Chan et al., 2020).

Human respiratory syncytial virus (hRSV) represents one of the most important causes of acute lower respiratory tract infection in young children and the elderly (Bohmwald et al., 2016; Canedo-Marroquín et al., 2017; Rey-Jurado and Kalergis, 2017; Carvajal et al., 2019). Regarding the neutrophil role during hRSV infection, it was shown that hRSV triggers NET release in human-derived neutrophils (Funchal et al., 2015; Cortjens et al., 2016; Muraro et al., 2018). Furthermore, NETs were observed in the airways and lungs of children with severe lower respiratory tract disease caused by hRSV (LRTD) (Cortjens et al., 2016). Furthermore, the extensive NET formation was associated with occluded airways of hRSV-infected calves, which may or not colocalize with viral antigens (Cortjens et al., 2016). These data suggest that NETs may or not trap viral particles, but their exacerbated formation during hRSV infection contributes to airway obstruction (Cortjens et al., 2016). Regarding the mechanism involved in such neutrophil response, it was shown that RSV induced the classical ROS-dependent NETosis in which viral particles are entrapped by DNA frameworks coated with MPO and NE. Furthermore, RSV-induced NETosis is also mediated by PAD-4-dependent histone citrullination and signaling through the PI3K/AKT signaling pathway (Muraro et al., 2018).

It was recently shown that during SARS-CoV-2 infection, the quantity of NETs release, measured as DNA-MPO complex, was increased in plasma, tracheal aspirate, and lung autopsies tissues from COVID-19 patients (Veras et al., 2020). Interestingly, this study also showed that infective SARS-CoV-2 but not the inactivated virus increased the release of NETs by neutrophils in an MOI-dependent manner (Veras et al., 2020). Notably, the release of NET after the SARS-CoV-2 challenge was abrogated when isolated neutrophils were pre-treated with a neutralizing anti-hACE2 antibody (αACE2) (Veras et al., 2020). Furthermore, the NET release was also prevented if neutrophils were pre-treated with the drug camostat, an inhibitor of the serine protease TMPRSS2 that blocks early interactions of SARS-CoV-2 S protein with the ACE2 receptor (Hoffmann et al., 2020). Furthermore, drugs also appear to inhibit viral replication as the viral load of neutrophils exposed to SARS-CoV-2 was reduced after αACE2 or camostat treatment (Veras et al., 2020).

This study also highlights the contribution of viral replication to the release of NETs upon the interaction of neutrophils with SARS-CoV-2. Incubation of neutrophils with tenofovir disoproxil fumarate (TDF), an RNA polymerase inhibitor (Clososki et al., 2020), reduced the release of NETs in neutrophils derived from healthy donors exposed to SARS-CoV-2. In addition, co-culture of SARS-CoV-2–activated neutrophils with epithelial cells promotes cell death *in vitro*, and this effect was prevented if DNAse was added in the culture medium (Veras et al., 2020). These data suggest an essential role for the extracellular DNA in promoting the cytotoxic effects of NETs. Together, these results underscore a possible detrimental role of NETs in the pathophysiology of COVID-19. Therefore, therapies targeted to inhibit the formation of NETs or promote the degradation of neutrophil extracellular DNA could be evaluated for a potential therapeutic benefit for COVID-19 (Veras et al., 2020).

Another study showed that high serum NETs, measured as cell-free DNA, DNA-MPO complex and citrullinated histones H3, are present in several hospitalized patients with COVID-19 (Zuo et al., 2020). The authors measure three different markers to assess the presence of NETs in blood corresponding to cell-free DNA, MPO-DNA, and Citrullinated Histone 3. Interestingly, sera from COVID-19 patients were a potent stimulator of NETs release when added to resting neutrophils, suggesting that a component present in serum may generate a pro-NETotic state on COVID-19 patients (Zuo et al., 2020).

It has also been described that those levels of plasma MPO-DNA complexes increased in intubated and dead COVID-19 patients (Middleton et al., 2020). The severity of the disease correlated directly with plasma MPO-DNA complexes. Soluble and cellular factors triggering NETs were significantly increased in COVID-19 patients. Furthermore, pulmonary autopsies showed NET-containing microthrombi with infiltrating neutrophils and platelets. Finally, neutrophils from COVID-19 patients displayed excessive NETs at baseline, and COVID-19 plasma triggered NET formation, blocked by neonatal NET-inhibitory factor (nNIF) (Middleton et al., 2020). Considering the prothrombotic clinical presentations of COVID-19 and the role of NETs in triggering such response points to targeting NETs as a novel therapeutic intervention for COVID-19 (Middleton et al., 2020).

Regarding additional pathways that regulate the generation of NETs during COVID-19, the pro-inflammatory cytokine IL-1β has been described as a critical inductor of NETs, both *in vivo and in vitro* assays (Meher et al., 2018). Furthermore, current evidence also suggests that NETs may promote the production of IL-1β precursors by macrophages that are used to amplify further the production of NETs (Hu et al., 2017). Under this scenario of excessive NET formation, alveolar and pulmonary endothelium becomes damaged, leading to the release of the von Willebrand factor (vWF), which activates blood platelets and neutrophils (Fernández-Pérez et al., 2021). Subsequently, activated platelets also stimulate neutrophils to produce NETs and clots, promoting airway obstruction impairing an efficient gaseous exchange (Pujhari et al., 2020).

IgA is another factor that can modulate NET formation during SARS-CoV-2 infection (Stacey et al., 2021). IgA is the second most abundant antibody present in the circulation and is enriched at mucosal surfaces. Therefore, this antibody plays a crucial role in protecting against mucosal pathogens, including viruses. IgA can also stimulate effector functions via the engagement of Fc alpha receptors (Fc-αRI) expressed on the surface of neutrophils (Stacey et al., 2021). In recent work, it was shown that IgA–virus immune complexes potentiate NETs release. This experiment used purified SARS-CoV-2 spike pseudotyped lentivirus, which were then opsonized with polyclonal IgA isolated from a convalescent COVID-19 donor serum. Interestingly virus opsonization increases the NET formation and potentiates a suicidal NETs release pathway. This process was independent of TLR signaling but required a functional NADPH oxidase complex. Therefore, targeting the NADPH oxidase complex may be a suitable strategy to decrease SARS-CoV-2 triggered NETs release (Stacey et al., 2021).

## Concluding Remarks

The mechanisms underlying NETs formation and their contribution to bacterial and viral infections have been studied as a primary function. However, during recent years, the role of NETs has changed, being an important matter when it comes to complications in several diseases. The different roles of NETs are in line with the fact that there is no specific pathway or stimuli to induce NETs release and that not all the stimuli are as good inducers of NETs as PMA or Gram-negative or -positive bacteria are. It is possible that the time of incubation and the dosing generates differences in the results obtained among the studies (Hoppenbrouwers et al., 2017). Also, as mentioned above, the antimicrobial properties of the granular enzymes of the NETs have been evaluated and, in some cases, do not generate a good antimicrobial capacity and probably have other immunomodulatory properties. Even more, the bactericidal capacities of NETs have been questioned because several studies have not evidenced a significant reduction of the initial inoculum in *in vitro* experiments (bactericidal effect). Further, the lysis (bacteriolytic effect) and the entrap of the bacteria (bacteriostatic effect) has not been consistently reproduced. In line with this, the citrullination of the DNA is also controversial because there are at least two more mechanisms, different from NETs release, that induce the citrullination: the leukotoxic hypercitrullination (LTH) which is not antimicrobial and can be induced by some virulence factors as toxins from *S. aureus* and *Streptococcus* spp., and the release of mitochondrial DNA due to a defect in mitophagy in neutrophils. Both processes are highly relevant in autoimmune disease such as rheumatoid arthritis and systemic lupus erythematosus, respectively (Konig and Andrade, 2016).

It is possible that the different types of NETs are induced at different time points during an infection, being possible that the first type of NETs release upon stimulation is composed of mitochondrial DNA, which still allows the survival of the cell. In this manner, the cell continues engulfing and performing antimicrobial properties. In addition, the release of mitochondrial DNA induces the secretion of type I interferon by plasmacytoid cells, generating a better immune response. Also, it is possible that NETs release composes of nuclear DNA occurs after the release of mitochondrial DNA, as an inflammatory consequence of the mitochondrial NETs release, inducing the NETosis process due to the nuclear NETs release. As example of this, the fibers that entrap the microorganisms are generated in vital NETs release, in opposite to the suicidal NETosis, were a cloud of DNA is generated, which not necessarily present antimicrobial properties (Yousefi and Simon, 2016).

It is currently studied that bacteria and viruses induce different pathways of NETs release, depending on the receptor activated by the microorganism. In this sense, bacteria have developed various mechanisms to evade the NETs release, generating a state of inflammation that allows pathogen spreading or the generation of a niche of infection—being the most common virulence factor among bacteria, the enzymes that degraded the NETs structure (Table 1). It is essential to mention that as NETs release, an inflammatory environment exists, and regulating this process is extremely necessary. One regulation occurs during the elimination of the NETs structure by the action of DNases, or C1q, allowing the recognition by the M1 macrophages (Farrera and Fadeel, 2013). Finally, it is crucial to find a proper definition of NETs due to the several aspects discussed above, which recognize if some pathogens induce or not the structure with antimicrobial properties or induce a structure that allows a higher inflammatory environment. Along these lines, this antimicrobial response will open targets for therapeutic intervention to treat diseases caused by bacteria and viruses, for example, the treatment of cystic fibrosis with DNases to liquify the sputum properly.

## Author Contributions

SB and BS: visualization. BS and OA: writing – origi nal draft. AK and SB: writing – review and editing. All authors contributed to the article and approved the submitted version.

## Conflict of Interest

The authors declare that the research was conducted in the absence of any commercial or financial relationships that could be construed as a potential conflict of interest.

## Publisher’s Note

All claims expressed in this article are solely those of the authors and do not necessarily represent those of their affiliated organizations, or those of the publisher, the editors and the reviewers. Any product that may be evaluated in this article, or claim that may be made by its manufacturer, is not guaranteed or endorsed by the publisher.
